# Association between intimate partner violence and child morbidity in South Asia

**DOI:** 10.1186/s41043-015-0016-y

**Published:** 2015-08-14

**Authors:** Elma Z. Ferdousy, Mohammad A. Matin

**Affiliations:** Department of Statistics, Jahangirnagar University, Savar, Dhaka

**Keywords:** Intimate partner violence, Childhood morbidity, South Asian Region, Bangladesh DHS, India DHS, Nepal DHS

## Abstract

**Background:**

This study investigates the association between intimate partner violence (IPV) against women and its impact on child morbidity in the south Asian region.

**Methods:**

The analysis uses logistic regression models with cross sectional nationally representative data from three countries - Bangladesh, India and Nepal. The data have been pooled from ‘Demographic and Health Surveys’ (DHS) of Bangladesh, Nepal and ‘National Family and Health Survey’ (NFHS) of India.

**Results:**

The study revealed that after controlling for potential confounders, children of mothers experiencing physical violence, sexual violence or both were more likely to have Acute Respiratory Infection (ARI) (OR_adj_ 1.57; 95 % CI 1.48–1.67), fever (OR_adj_ 1.44; 95 % CI 1.35–1.54) and diarrhea (OR_adj_ 1.56; 95 % CI 1.44–1.69).

**Conclusions:**

The results highlight that IPV can influence childhood morbidity and support the need to address IPV with a greater focus within current child nutrition and health programs and policies.

## Background

During the last decade several studies investigated the impact of intimate partner violence (IPV) on children’s health. A recent study by World Health Organization (WHO) shows that the global prevalence of physical and/or sexual intimate partner violence among all ever-partnered women was 30.0 % [39]. The estimates of physical and sexual violence are substantially higher in the developing countries in general [12, 14, 25]. South Asia reports some of the highest rates of violence [17, 20, 30].

Evidence is available on negative consequences of IPV for women’s health and well-being [6]. Several studies reported the link between IPV and women’s mental health and depression [8, 28]. Some of the studies also highlighted an association between such violence and reproductive health outcomes like non-use of contraception, gynecological problems, unintended pregnancy and sexually transmitted infections including HIV/AIDS. These results suggest there may be direct or indirect impact of IPV on children as well.

Significant attention has also been dedicated to the potential negative role of IPV on the health and survival of infants. Some population based studies have reported considerable adverse effects of IPV upon gestational and birth outcomes [3]. This also contributes in part to the association between IPV and prematurity or low birth weight [31, 38].

Studies on the association between IPV and childhood mortality are much more limited. Most of such studies come from developed countries. Scanty literature on this topic in the developing world includes some studies from India and one study from Bangladesh. Several studies from north India suggested significant association between lifetime IPV and infant mortality risks [1, 2, 19, 23]. A population-based study from rural Bangladesh convincingly showed association between IPV and child mortality [5].

Studies in Bangladesh convincingly showed that children of women exposed to IPV have greater exposure to potentially dangerous conditions like diarrhea and Acute Respiratory Infection (ARI) [4, 36]. Impact of IPV on child nutrition has also been demonstrated by a few studies [3, 40].

The current study builds upon the existing literature and explores association between IPV and childhood morbidity expanding the study beyond a single country to include three neighboring countries in South Asia, i.e., Bangladesh, India and Nepal.

## Methods

### Study population and sampling procedure

The study uses three datasets [16]– Bangladesh Demographic and Health Survey (DHS) 2007, India National Family and Health Survey (NFHS) – 2005–06 and Nepal DHS 2011. Both DHS and NFHS are nationally-representative household surveys on population, health, and nutrition. These surveys employed a two stage sampling procedure with the first stage involving the selection of primary sampling units (PSUs); these were probability proportional to the size and represent the number of households within the PSU. The second stage used a systematic technique to sample households from each of the selected PSUs. The details of the methods and procedures used in data collection in DHS and NFHS surveys are provided in the respective DHS and NFHS reports [18, 27, 29]. Informed consent was obtained from the study participants. All these three surveys have similar modules on violence. These questions were administered to a sub-sample.

This secondary analysis includes a total of 40,394 women from India, Bangladesh and Nepal. The women selected for the violence module had at least one child under the age of 5 years during the survey. We included all the children under the age of 5 years in this sample. Therefore a total number of 58,725 children of these women were included in the study.

### Ethical clearance and considerations

The original survey was administrated in accordance with the WHO ethical and safety guidelines for research on IPV. The institution review board of ICF Macro (formerly Macro International Inc.), an advisory, implementation, and evaluation services firm providing research-based solutions to U.S. federal government agencies in health and other areas. Reviewed and approved the surveys used in the study. The interviewers received special training to implement this module. The training focused on how to ask sensitive questions, ensure privacy, and build rapport between interviewer and respondent. Verbal Informed consent was also obtained from the respondents. Details of the informed consent statement can be found in the appendix section of the respective reports [18, 27, 29].

### Exposure and outcome measures

This study considers IPV as the main exposure variable while child morbidity as the outcome of interest.

### IPV

Women were asked the following questions about their experience of IPV involving their husbands over the past 12 months.

i. Has your husband pushed, shook or threw something?
ii. Has your husband slapped you?
iii. Has your husband twisted your arm or pulled your hair?
iv. Has your husband punched with fist or something harmful?
v. Has your husband kicked you, dragged you or beaten you up?
vi. Have you been threatened by a weapon or had a weapon used against you by your husband? Has your husband been choked you or burn you on purpose?
vii. Have you been physically forced to have sex or otherwise sexually abused by your husband?

A new dummy IPV variable was created based on the answer to the above questions. A positive response to any one of the first six questions was treated as indicative of physical IPV victimization and was coded as “1” and if none of the responses was positive, exposure to physical violence was coded as “0”. A similar strategy was followed for deriving a variable indicating exposure or non-exposure to sexual IPV. Then two more variables were created to indicate exposure to any IPV or both IPV.

### Child morbidity

Women were asked whether their child had been ill with fever, diarrhea, or a cough accompanied by short, rapid breathing in the 2 weeks prior to the surveys. Acute Respiratory Infection (ARI) was defined as the presence of cough, along with short, rapid breathing. Binary variables for Diarrhea, ARI and Fever were created which indicated the presence of each of these outcomes among the children in the past 2 weeks prior the survey. For these cases, “0” indicated absence of the symptoms while “1” indicated presence of the same.

### Covariates

The study controlled for well established correlates of child morbidity such as age, religion, education, living standard, employment status and areas of residence [18, 27, 29].

Respondent’s age was classified into 5 groups. The respondents were asked if they attended any educational institution and if yes then the highest level completed (primary, secondary, higher) was asked. We used the information to classify education of the mother into the following categories: (no education, primary, secondary & higher).

Respondents were asked about the main source of drinking water for their household and presented with options like piped water, tube well or borehole, dug well, spring water, surface water etc. The responses were grouped into three major categories: Tube well or borehole, piped water and other sources. Women were asked about the toilet facility and the responses were classified in the groups of pit latrine, flush and no facility. Cooking fuel of the household was classified into: electricity, gas, kerosene, wood/coal and others.

We used the DHS wealth index calculated as follows: each asset was assigned a weight (factor score) generated through principal component analysis, and the resulting asset scores were standardized in relation to a normal distribution with a mean of zero and standard deviation of one. Each household was then assigned a score for each asset, and the scores were summed for each household; individuals were ranked according to the total score of the household in which they resided. The sample was then divided into quintiles from one (lowest) to five (highest).

### Statistical analysis

We calculated descriptive statistics for socio-demographic and morbidity characteristics for our selected sample individuals. The relationhip between morbidity and selected demographic and socio-economic factors were examined first using chi-square test. Then we used logistic regression models for exploring the relationship between IPV and different child morbidity outcomes. After this step we ran multiple logistic regression analysis. We calculated the adjusted odds ratio to measure the strength of associations. SPSS version 17 was used for the analysis.

## Results

Background characteristics of the women and their children are presented in Table 1. Most of the women were aged between 20 and 30 years and around 40 % of them had no formal education. Approximately one third of these women reported experience of IPV.

**Table 1:** Background characteristics of women and their children included in the study (pooled data)

Variables	Percent of Women (*n* = 40,394)	Variables	Percent of Children (*n* = 58,725)
Age Groups		Mother’s Age	
15–19	9.5	15–19	5.2
20–24	24.8	20–24	32.3
25–29	30.4	25–29	34.6
30–39	28.6	30–39	25.4
40–49	6.8	40–49	2.5
Education Level		Mother’s Education	
No Education	38.6	No Education	40.8
Primary	37.5	Primary	37.1
Secondary	15.6	Secondary	14.5
Higher	8.3	Higher	7.6
Type of residence		Type of residence	
Urban	39.6	Urban	37.8
Rural	60.4	Rural	62.2
Wealth Index		Wealth Index	
Poorest	17.5	Poorest	17.8
Poorer	17.8	Poorer	18.6
Middle	19.8	Middle	20.7
Richer	21.7	Richer	21.9
Richest	23.3	Richest	21.0
Source of Drinking Water		Source of Drinking Water	
Tube well or Borehole	32.1	Tube well or Borehole	36.6
Piped Connection	41.1	Piped Connection	38.8
Other	26.8	Other	24.6
Toilet Facility		Toilet Facility	
No Facility	41.6	No Facility	30.8
Flush	28.2	Flush	26.5
Pit latrine or other	30.2	Pit latrine or other	42.7
Exposed to IPV		Morbidity	
Physical violence	30.4	Fever	15.4
Sexual violence	8.8	Diarrhea	9.8
Both/Either	32.6	ARI	8.9

In Table 2, presents the results of cross tabulation between different form of violence against women and the three diseases of interest. Across board a higher proportion of the children of abused mothers experienced the illnesses. Mothers exposed to sexual violence reported more incidents of child fever, diarrhea or ARI which are 22.6, 14.9 and 18.0 % respectively compared to children of sexually non- abused mothers 14.3, 8.3 and 13.2 % respectively (*p* < 0.001).

**Table 2:** Different forms of violence and child morbidity

	Fever		Diarrhea		ARI	
	No	Yes	No	Yes	No	Yes
Sexual Only						
No	85.7 %	14.3 %	91.7 %	8.3 %	86.8 %	13.2 %
Yes	77.4 %	22.6 %	85.1 %	14.9 %	82.0 %	18.0 %
p value	<0.001		<0.001		<0.001	
Physical Only						
No	86.9 %	13.1 %	92.4 %	7.6 %	90.9 %	9.1 %
Yes	80.6 %	19.4 %	88.3 %	11.7 %	76.1 %	23.9 %
p value	<0.001		<0.001		<0.001	
Both						
No	85.6 %	14.4 %	91.6 %	8.4 %	88.2 %	11.8 %
Yes	76.5 %	23.5 %	84.4 %	15.6 %	60.7 %	39.3 %
p value	<0.001		<0.001		<0.001	

Table 3 shows the results of multivariate logistic regression analysis controlled for mothers’ age, education, and wealth index, type of residence, source of drinking water, toilet facility and type of cooking fuel as covariates. Table 4 shows adjusted and unadjusted result for each of the controlling variables.

The results show that Maternal experience of physical IPV increased the likelihood of child fever by 46 % (AOR: 1.46, 95 % CI: 1.37–1.55), sexual IPV by 67 % (AOR: 1.67, 95 % CI: 1.59–1.87), and both physical and sexual IPV by 64 % (AOR: 1.64, 95 % CI: 1.49–1.81).

**Table 3:** Adjusted odds ratios and 95 % confidence intervals for associations between different forms of maternal IPV and under-five child morbidity (pooled data)*

	Fever	Diarrhea	ARI
Physical IPV only			
No	1.00	1.00	1.00
Yes	1.46 (1.37, 1.55)	1.55 (1.46, 1.64)	1.53 (1.42, 1.64)
Sexual IPV only			
No	1.00	1.00	1.00
Yes	1.67 (1.59, 1.75)	1.79 (1.62, 1.98)	1.72 (1.59, 1.87)
Both physical and sexual IPV			
No	1.00	1.00	1.00
Yes	1.64 (1.49, 1.81)	1.66 (1.49, 1.81)	1.76 (1.61, 1.92)
Physical or sexual IPV			
No	1.00	1.00	1.00
Yes	1.44 (1.35, 1.54)	1.56 (1.44, 1.69)	1.57 (1.48, 1.67)

*Adjusted for maternal age, maternal education, type of residence, types of cooking fuel, wealth index, toilet facility, and source of drinking water

**Table 4:** Adjusted and unadjusted odds ratios and 95 % confidence intervals any IPV and covariates

	Fever				Diarrhea				ARI			
	Unadjusted		Adjusted		Unadjusted		Adjusted		Unadjusted		Adjusted	
Variables	OR	CI	OR	CI	OR	CI	OR	CI	OR	CI	OR	CI
Any IPV												
No (Reference)	1		1		1		1		1		1	
Yes	1.61	1.51	1.44	1.35	1.75	1.62	1.56	1.44	1.74	1.64	1.57	1.48
		1.71		1.54		1.88		1.69		1.84		1.67
Mother’s Age												
15–19 (Reference)	1		1		1		1		1		1	
20–24	1.95	1.74	1.94	1.73	1.88	1.65	1.82	1.58	1.93	1.73	1.9	1.71
		2.19		2.18		2.19		2.11		2.14		2.12
25–29	1.71	1.53	1.76	1.57	1.55	1.35	1.52	1.32	1.64	1.48	1.68	1.51
		1.91		1.98		1.78		1.76		1.83		1.88
30–39	1.43	1.26	1.48	1.31	1.27	1.09	1.27	1.09	1.27	1.13	1.31	1.17
		1.61		1.67		1.47		1.48		1.41		1.47
40–49	1.14	0.98	1.15	0.99	0.90	0.78	0.89	0.73	0.97	0.85	0.99	0.86
		1.31		1.33		1.09		1.07		1.12		1.14
Mother’s highest education level												
No Education (Reference)	1		1		1		1		1		1	
Primary	1.14	1.06	1.08	1.00	0.95	0.86	0.93	0.84	1.22	1.13	1.12	1.04
		1.24		1.18		1.05		1.03		1.31		1.20
Secondary	0.98	0.92	1.04	0.97	0.92	0.85	0.99	0.90	1.05	0.99	1.09	0.96
		1.05		1.12		1.00		1.08		1.12		1.24
Higher	0.77	0.69	0.98	0.85	0.60	0.51	0.78	0.65	0.82	0.74	0.95	0.88
		0.86		1.12		0.69		0.94		0.91		1.02
Type of Residence												
Urban (Reference)	1		1		1		1		1		1	
Rural	1.15	1.09	1.03	0.95	1.12	1.04	1.06	0.93	1.10	1.04	1.16	1.07
		1.22		1.14		1.20		1.18		1.16		1.25
Wealth Index												
Poorest (Reference)	1		1		1		1		1		1	
Poorer	1.05	0.96	0.99	0.91	0.95	0.85	0.95	0.85	1.05	0.97	0.99	0.91
		1.14		1.09		1.06		1.07		1.14		1.08
Middle	0.91	0.84	0.88	0.80	0.94	0.85	0.96	0.86	0.98	0.90	0.92	0.83
		1.00		0.97		1.05		1.08		1.06		1.00
Richer	0.87	0.80	0.91	0.81	0.84	0.75	0.89	0.77	0.90	0.83	0.86	0.77
		0.95		1.02		0.93		1.02		0.98		0.96
Richest	0.70	0.64	0.86	0.75	0.62	0.56	0.74	0.62	0.71	0.66	0.78	0.68
		0.76		1.00		0.70		0.89		0.77		0.89
Source of Drinking water												
Tube well/ Borehole (Reference)	1		1		1		1		1		1	
Piped Water	0.98	0.92	0.80	0.68	0.78	0.72	0.98	0.90	1.03	0.96	0.91	0.77
		1.05		0.94		0.85		1.07		1.10		1.07
Other	1.51	1.41	1.45	1.34	1.49	1.39	1.32	0.70	1.05	0.96	1.43	1.33
		1.62		1.56		1.58		2.47		1.14		1.53
Type of Toilet Facility												
Pit Latrine (Reference)	1		1		1		1		1		1	
Flush	0.95	0.90	0.95	0.74	0.79	0.73	0.83	0.68	0.97	0.92	0.91	0.57
		1.01		1.20		0.85		1.02		1.03		1.44
No facility	1.56	1.45	1.79	1.50	1.69	1.56	1.63	1.19	1.03	0.93	0.94	0.74
		1.69		2.13		1.80		2.24		1.15		1.19
Type of Cooking fuel												
Electricity (Reference)	1		1		1		1		1		1	
Gas	1.33	1.00	0.80	0.60	1.29	0.70	1.05	0.95	1.32	0.84	0.86	0.65
		1.77		1.08		2.40		1.17		2.07		1.13
Kerosene	1.39	1.09	1.27	0.99	1.36	1.01	1.10	0.88	1.31	1.05	1.19	0.95
		1.76		1.62		1.83		1.37		1.63		1.49
Wood/Coal	1.73	1.1	1.14	0.71	1.45	1.02	1.26	0.87	1.43	1.10	1.6	1.47
		2.74		1.81		2.06		1.82		1.85		1.73
Other	1.96	1.55	1.75	1.61	1.78	1.32	1.61	1.19	1.78	1.44	1.58	1.34
		2.48		1.90		2.38		2.18		2.21		1.89

Maternal exposure to physical IPV increased the risks of diarrhea of the child by 55 % (AOR: 1.55, 95 % CI: 1.46–1.64), sexual IPV by 79 % (AOR: 1.79, 95 % CI: 1.62–1.98) and both physical and sexual IPV by 66 % (AOR: 1.66, 95 % CI: 1.49–1.81).

Maternal experience of physical IPV increased the likelihood of child’s ARI by 53 % (AOR: 1.53, 95 % CI: 1.42–1.64) sexual IPV by 72 % (AOR: 1.72, 95 % CI: 1.59–1.87) and both physical and sexual IPV by 76 % (AOR: 1.76, 95 % CI: 1.61–1.92).

## Discussion

The central aim of this study was to investigate the association between Intimate Partner Violence against women and child morbidity in South. The results of the study provide evidence of an association between IPV and child morbidity in these three countries of South Asia. The results are in line with previous findings [1, 2, 5, 15, 19, 21, 23, 33, 35, 36]. We found that a higher proportion of the children of IPV survivors reported fever, diarrhea or ARI compared to the non abused mothers irrespective of the form of violence. There may be several approaches to explain the association between IPV and child morbidity. We offer the following three classes among them:

- Poor mental health of the mother: Women who experience IPV tend to have higher level of psychological stress [10, 26] which may impede their capacity to provide adequate child care. There are physiological reasons for psychological stress is associated to anemia. Psychological stress is a risk factor for oxidative stress [9, 13], that produce free radicals and other organic molecules capable of damaging living tissue and can destroy red blood cells prematurely that results in a potential cause of hemolytic anemia. Additionally chronic stress has been found to result in long term reduction of hemoglobin suggesting that stress interferes with protein synthesis required to create new red blood cells. Conditions like anemia can weaken a person and lowers a person’s productivity through a decreased ability to work [37]. These physical conditions contribute poor child caring ability of a woman.
- Lack of empowerment: Intimate partner violence is often used as a tool for controlling women [32]. Thus IPV is also strongly associated with inability of a woman to make decision for herself and for her children [34] with obvious implications for her own health and nutrition and that of her children. Lack of empowerment also limits mother’s ability to access household resources, health care services and participation in common health care activities [32, 34] which in turn lowers their ability to provide appropriate care to their children.
- Poor physical condition of the mother: In case of extreme IPV often the victims become physically unfit to give proper care to their children.

Besides, the children who witness IPV themselves tend to experience psychological stress; which in turn result in poor physiological conditions making the children vulnerable to morbidity [7, 11, 22]. Investigation of the specific pathways through which IPV might influence child health and morbidity should be a high priority for future research.

### Limitation

The cross sectional study design represents a limitation of this study. Though there may be some concern regarding the temporal ordering of IPV and child morbidity in cross sectional studies, previous research shows that IPV in this subcontinent is a relatively stable phenomenon that has its root in patriarchal structures and community norms of high acceptability of wife beating [24].

Another limitation of the study is that both the outcome and the exposure variables are measured through self reports. Although in the DHS much care and preparation is taken into the design and execution of the interviews to create a safe atmosphere in which respondents would feel comfortable discussing the IPV, high stake in disclosure may always cause under reporting.

## Conclusion

The study shows that IPV negatively affects child’s health not only in individual countries, but also in the South Asia region. The children of an abused mother are more likely to experience diseases like fever, diarrhea and ARI compared to the children of non-abused mothers. IPV should be considered as a major health problem affecting not only women, but also their children in South Asia. Although it seems IPV is most strongly associated with socioeconomic demographic and contextual disadvantages, incidents of IPV among socially marginalized groups may aggravate the negative effects of cumulative disadvantages of poor child health outcomes. The health sector in these countries needs to take action to eliminate IPV for improving child health. Future studies should investigate the influence of potential mechanisms mediating the association of IPV and child morbidity.
